# Identification of a Novel Hepatitis E Virus Genotype 3 Strain Isolated from a Chronic Hepatitis E Virus Infection in a Kidney Transplant Recipient in Switzerland

**DOI:** 10.1128/genomeA.00345-17

**Published:** 2017-05-18

**Authors:** Bo Wang, Dominik Harms, Jörg Hofmann, Diana Ciardo, Agnes Kneubühl, C.-Thomas Bock

**Affiliations:** a Department of Infectious Diseases, Robert Koch Institute, Berlin, Germany; b Institute of Medical Virology, Charité University Medicine, Berlin, Germany; c Labor Berlin, Charité-Vivantes GmbH, Berlin, Germany; d Corelab Immunology, Viollier AG, Allschwill, Switzerland; e Department of Internal Medicine/Nephrology, Spital Lachen, Lachen, Switzerland

## Abstract

Hepatitis E virus genotype 3 (HEV-3) is the causal pathogen of chronic hepatitis E. We report here the full-length genome sequence of an HEV-3 strain, isolated from a kidney transplant recipient in Switzerland (SW/16-0282). This HEV-3 strain showed less than 88% homology compared to known strains, suggesting a new HEV-3 strain.

## GENOME ANNOUNCEMENT

Hepatitis E virus (HEV) is a single-stranded RNA virus and a common cause of acute hepatitis (1, 2). Four main HEV genotypes, as well as the recently reported HEV genotype 7, infecting humans have been described. HEV genotypes 1 and 2 have been linked to waterborne outbreaks in developing countries that exclusively infect humans. In contrast, HEV genotypes 3, 4, and 7 are predominant in industrialized countries, while zoonotic transmission from animal reservoirs to human has been suggested (3, 4). It is well established that prolonged HEV viremia and chronic hepatitis E with HEV genotype 3 (HEV-3) can occur in immunocompromised patients (5, 6). Recent studies revealed an HEV-seroprevalence among blood donors (4.9%) and chronic infections in patients with HIV (2.6%) in Switzerland. However, HEV seroprevalence in Switzerland is considerably lower than in other European countries, which may be explained by the strict regulation of animal and meat imports (7–9). Here, we report the full-length genome sequence of an HEV-3 strain (SW/16-0282), isolated from a kidney transplant recipient with chronic hepatitis E in Switzerland.

The virus was isolated from a 58-year-old male kidney transplant recipient from Lachen, Switzerland. The patient presented clinically with hepatitis symptoms and moderate elevated liver enzymes in June 2016 while being treated with immunosuppressive drugs. The first test for HEV IgM/ IgG was negative and hepatitis was considered because of drug toxicity. No recovery of hepatitis was seen despite changing drugs. In August 2016, a diagnosis of hepatitis E relied on a further increase of liver enzyme levels and detection of anti-HEV IgM/IgG and HEV RNA in serum (3.1 × 10E+5 IU/mL) and feces (2.7 × 10E+7 IU/mL stool suspension). The HEV IgG/IgM immunoassay (recomLine, Mikrogen, Germany) showed a clear response to the C-terminal part of the capsid antigen (O2C; genotype 3). However, there was no evidence for a specific HEV-3 strain. Nutritional regimens limited the patient’s meat consumption to beef and fish (no venison and swine).

Viral RNA extraction from patient serum and feces was performed using the High Pure Viral nucleic acid kit (Roche Diagnostics, Germany) according to the manufacturer’s instructions. After cDNA synthesis using Transcriptor first-strand cDNA synthesis (Roche Diagnostics, Germany), the complete viral genome was amplified using KAPA HiFi HotStart ReadyMix PCR (Kapa Biosystems, USA). The 5′ and 3′ sequences were determined using 5′ and 3′ rapid amplification of cDNA ends (Roche Diagnostics, Germany). HEV amplicons were sequenced with the BigDye Terminator version 3.1 cycle sequencing kit (Applied Biosystems, USA) in both directions. Whole-genome sequence and phylogenetic analyses were done using Geneious version 10.0.5 and MEGA7 software (10).

The complete genome of SW/16-0282 is 7,222 nucleotides in length, excluding the poly(A)-tail, with a G+C content of 56% harboring the expected HEV open reading frames (ORFs) with 1,703 (ORF1), 660 (ORF2), and 122 (ORF3) amino acids, respectively. Phylogenetic reconstructions based on the whole-genome sequences demonstrated that SW/16-0282 belongs to HEV-3. SW/16-0282 shared the highest homology with the HEV subgenotype 3h TR19 strain (87.8%; JQ013794). Because of the poor homology to any HEV-3 subgenotype, it must be verified by comprehensive genetic analyses of HEV strains from human and animal reservoirs whether the SW/16-0282 strain represents a new HEV subgenotype 3l.

### Accession number(s).

The complete genome sequence of SW/16-0282 has been deposited in GenBank under the accession number KY780957.
